# Bringing Attention to a Forgotten Age Group: A Discussion on Late-Onset Bipolar Disorder

**DOI:** 10.7759/cureus.83904

**Published:** 2025-05-11

**Authors:** Sanyukta Savant, Krishna Geeth Chirumamilla, Luba Leontieva

**Affiliations:** 1 Medicine, Independent Research, Somerville, USA; 2 Psychiatry, State University of New York Upstate Medical University, Syracuse, USA

**Keywords:** atypical anti-psychotic, bipolar affective disorder, bipolar disorder- 1, depressive episode, geriatric psychiatric perspective, late-onset bipolar disorder, manic episodes, older-age bipolar disorder, unspecified bipolar disorder

## Abstract

Affecting millions worldwide, bipolar disorder is a chronic condition most commonly diagnosed before the age of 50. However, diagnosing bipolar disorder beyond this age bracket presents unique challenges and is referred to as late-onset bipolar disorder. Due to the limited availability of data, this diagnosis remains complex.

Although several efforts have been made to understand the impact of age of onset and neurologic etiologies on the condition, extensive research on this subject is unavailable, which will definitely facilitate enhancing services to this group.

We present the case of a 75-year-old male with a past psychiatric history of late-onset bipolar disorder, anxiety, depression, and a historical diagnosis of obsessive-compulsive personality disorder. He was first diagnosed with bipolar I disorder in his 60s following a manic episode by his primary care physician. His condition was subsequently managed with lamotrigine 100 mg daily and olanzapine 2.5 mg daily. No prior hospitalizations for mania or any other psychiatric etiology were noted.

During this admission to the hospital, the patient presented with symptoms of mania and paranoia and was difficult to redirect. He exhibited signs of distractibility, tangentiality, pressured speech with increased pace, flight of ideas, and reduced need for sleep.

This case helps highlight the variability in presentation of bipolar disorder in older adults, unexplained by other organic causes, and its successful management. It also instigates a discussion about limited data and guidelines in this age group.

## Introduction

Bipolar and related disorders encompass a range of conditions, including: bipolar I disorder (BD-I), bipolar II disorder (BD-II), cyclothymic disorder, other specified bipolar and related disorders, and unspecified bipolar or related disorders. Older age bipolar disorder (OABD) is a term that is used to describe bipolar disorder (BD) occurring among individuals ≥50 years of age [1].

OABD is an umbrella term, with two main categories: early-onset bipolar disorder (EOBD) and late-onset bipolar disorder (LOBD). These two categories can be distinguished by their age of onset: EOBD - patients who have their first episode before 50 years of age, and LOBD - patients who have their first episode at the age of 50 or older. While patients with EOBD are more likely to have classic manic episodes with higher prevalence of psychotic features, LOBD is more likely to present with depressive or mixed states rather than classic mania. This classification helps distinguish and tailor management approaches. A discussion of LOBD cases is crucial, as the data on bipolar disorder in older adults is limited [2].

To diagnose BD-I, per the General DSM-5 Diagnostic Criteria for Bipolar and Related Disorders, an individual must meet the criteria for a manic episode. While this manic episode may be preceded by or followed by hypomanic or major depressive episodes, these are not required for the diagnosis. A manic episode is defined as a distinct period of at least seven consecutive days (or any duration if hospitalization is necessary) characterized by persistently elevated or irritable mood and increased activity or energy. A hypomanic episode is defined as a distinct period of persistently elevated or irritable mood with increased activity or energy lasting for at least four consecutive days [1]. And to qualify as a manic or hypomanic episode, at least three of the following symptoms must be present (4 if the mood is primarily irritable): inflated self-esteem or grandiosity, reduced need for sleep, increased talkativeness, racing thoughts or flight of ideas, high distractibility, increased goal-directed activity or psychomotor agitation, and engagement in risky behaviors with potential negative consequences [1].

It's important to note that these symptoms should not be attributed to substance use or medical conditions. Furthermore, a manic episode is significantly more severe than a hypomanic episode and results in marked impairment in social or occupational functioning or necessitates hospitalization [1].

The DSM-5 also includes an unspecified bipolar disorder category. This applies to presentations where symptoms characteristic of bipolar disorder cause significant distress or impairment but don't meet the full criteria for any specific bipolar disorder. This category is used when the clinician chooses not to specify why the criteria aren't fully met or when there's insufficient information for a more specific diagnosis. This allows for clinical flexibility in diagnosing bipolar-like presentations that don't fit neatly into other categories [1]. Based on the symptoms of this patient, the diagnosis of unspecified bipolar disorder seemed more fitting.

Epidemiological studies suggest a lifetime prevalence of around 1% for bipolar type I in the general population. A large cross-sectional survey of 11 countries was conducted and found the overall lifetime prevalence of bipolar spectrum disorders to be 2.4%, with a prevalence of 0.6% for bipolar type I and 0.4% for bipolar type II. Evidence indicates that OABD accounts for a quarter of all cases of BD [3].

The age at which the illness begins can heavily influence its characteristics and progression. The International Society for Bipolar Disorder (ISBD) Task Force on Older-Age Bipolar Disorder (OABD), in 2015, presented the first report stating that “In the coming generation, older adults with bipolar disorder (BD) will increase in absolute numbers as well as proportion of the general population” [2].

This case report aims to bring awareness to the varied presentations of late-onset bipolar disorder and initiate a discussion to further conduct research on this topic. This chronic condition imposes substantial burdens on the patients, their families, and public health systems. 

Here, we present the case of a 75-year-old male with a past psychiatric history of late-onset bipolar disorder, with the first manic episode documented at the age of 66. He was admitted to the hospital following an episode of physical aggression toward his wife and verbal threats made to a family member. This case holds significance, as it discusses older age bipolar disorder, with no clear organic causes, and addresses the scarcity of literature for treatment guidelines for OABD patients. With a significant number of patients experiencing late-onset bipolar disorder, a discussion needs to follow.

## Case presentation

The patient is a 75-year-old retired mechanical designer and Korean War veteran with a nine-year history of late-onset bipolar I disorder diagnosed by his primary care physician. His past psychiatric history includes anxiety, depression, and a historical diagnosis of obsessive-compulsive personality disorder, though his chart review provides limited information. At the time of his hospital presentation, he was taking lamotrigine 100 mg daily and olanzapine 2.5 mg daily. His records also indicate a brief use of paroxetine in 2012 and buspirone in 2013, with medications dispensed for two years, although the indications for their use remain unclear. No prior psychiatric hospitalizations were noted.

According to the information collected from collateral sources, the patient had been symptomatically stable two to three months before his presentation to the hospital. However, during this period, he experienced gradual decompensation of symptoms, marked by impulsive purchases, planning unexpected trips, and manic behavior. He engaged in continuous work on projects at odd hours of the day and justified his reduced need for sleep by describing himself as an “active person”. Additionally, he displayed physical aggression toward his sister and wife. While the patient himself denied any physical aggression toward his wife, collateral information revealed that during a verbal altercation, he pushed her against the couch multiple times, then against garbage cans, and grabbed her by the hair in their garage. Furthermore, he made threats to his son's ex-wife, whom he disapproved of. These conflicts stemmed from various stressors within their respective relationships, and the conflict with his wife was in relation to a very remote past incident of infidelity. These incidents raised significant safety concerns for his family and ultimately resulted in his hospitalization after a pickup order issued by his primary care doctor at the request of his son. When these behaviors were brought up with the patient, he initially seemed dismissive and minimized the impact his actions had on his family, and blamed his wife and son for his outburst.

According to the information obtained from the patient and collateral, the patient experienced uncontrollable anger, triggered by a longstanding unresolved argument between his wife and him, and "lack of closure." According to the patient, his wife had an incident of infidelity, very early on in their marriage, which was never properly addressed. Before the argument with his wife, the conflict resurfaced when the patient saw the name of the person with whom his wife was in a relationship with in an obituary. This escalation in emotions led to a series of concerning behaviors that raised safety concerns for his family and ultimately resulted in his hospitalization. Per collateral information, for a few days before the argument, the patient exhibited signs such as decreased need for sleep, working on projects in the middle of the night, like building a birdhouse, and having hard-to-interrupt speech on topics that he usually did not talk about. He also planned a trip to visit his siblings, whom he hasn’t seen in many years. He made threats to harm his son’s ex-wife while mentioning purchasing firearms.

On initial mental status examination, the patient appears well-groomed and dressed in hospital scrubs, maintaining good hygiene. Throughout the interview he was cooperative but angry, making appropriate eye contact without any signs of psychomotor agitation or retardation. His speech was pressured, with increased rate and quantity, jumping from one idea to another without clear connections, indicative of flight of ideas. The patient was hard to interrupt at times, focusing on telling ‘his side of the story’. His mood was described as "okay," and his affect was dysphoric. He exhibited mood lability and was intermittently tearful and angry at the treatment team during the interview. His thought process was largely tangential, veering off-topic and occasionally circumstantial, making it hard to follow. He showed significant preoccupation with the incident leading to his admission, expressing both guilt and anger, while attempting to justify his actions. He needed multiple redirections to answer the questions that were posed to him, resisting attempts to refocus on relevant questions. He endorsed paranoia and ideas of persecution, reporting that his son was trying to get him involuntarily admitted to the hospital and was in some way plotting against him but was unable to reasonably elaborate on his son’s motivation for doing that. There were no perceptual disturbances, such as hallucinations, noted. The patient was oriented to time, place, and person, with intact attention and memory. He demonstrated poor insight into his condition. Although he described his current state as manic, he didn’t seem to have a good understanding of his condition. He displayed poor judgment, not acknowledging the impact of his actions or understanding the consequences. A diagnosis of unspecified bipolar disorder was placed due to its atypical presentation. This diagnosis is used when symptoms cause significant distress or impairment but fail to meet the full criteria for a defined bipolar disorder [1].

Family history

The patient is the youngest of the three siblings and had a turbulent childhood. Although his father was cordial toward him, he witnessed verbal and physical abuse toward his mother and other siblings. The patient was reported to have been verbally abusive toward his wife for many years, though no instances of physical abuse were reported until his recent physical altercation. He rarely kept in touch with his siblings, making his decision to plan a surprise trip to visit his brother all the more unexpected. Per collateral information, his mother was talkative and hard to interrupt, but no official diagnosis of bipolar was ever made. Medically, she was diagnosed with dementia later in life. The patient did not report any formally diagnosed mental illnesses in first-degree relatives.

Past history

The patient experienced several stressors early in life, including 20 months of military service in Korea, where he was exposed to Agent Orange. According to the patient, upon returning from Korea, he noticed significant changes in his personality, and referred to himself as “self-assured and with an attitude”. He doesn’t mention any trauma during his years of service, but it is possible that there is a minimization of symptoms, as it's been more than 50 years since returning from Korea. Diagnosis of PTSD has been ruled out due to a lack of symptoms.

The patient provided a history of a car accident in 2014, in which his car was T-boned at an intersection. He was not wearing a seatbelt and was partially ejected from the car, which resulted in brief loss of consciousness, a concussion, and a fractured T5 vertebra. Following this accident, he experienced his first manic episode, during which he made impulsive purchases of buying an expensive car, which he referred to as his “retirement car,” and a house for his son. He was diagnosed with bipolar I disorder by his primary care physician that same year. His condition was subsequently managed with lamotrigine 100 mg daily and olanzapine 2.5 mg daily, which he was taking at the time of the hospitalization. The exact history is unclear, but his chart review reveals that the patient was historically taking paroxetine in 2012 and buspirone in 2013. Records indicate that the medications were dispensed for two years, with no subsequent refills. However, the patient did not report any clear history of depression or its management.

A non-contrast CT scan of the head was conducted, which was negative and inconclusive for acute intracranial pathology such as hemorrhage, ischemia, or mass effect. His medical history also includes three concussions during his teenage years while playing football.

The patient’s medical history includes benign prostatic hyperplasia (BPH), managed with tamsulosin 0.4 mg nightly; arthritis and degenerative joint disease affecting multiple joints; gastroesophageal reflux disease (GERD), managed with esomeprazole 40 mg daily; glaucoma; right bundle-branch block (RBBB); sinus node dysfunction and paroxysmal supraventricular tachycardia (PSVT); and neuropathic pain, for which he takes gabapentin 300 mg three times daily. He also takes verapamil 120 mg (24-hour formulation) nightly for cardiac indications.

Social history

The patient met his wife during college and got married at an early age. He earned a professional degree and soon after began working as a professional designer for a company. While expecting his first child, he was deployed to Korea during the conflict. He had to leave his job and family, which he expressed was stressful. Upon returning, he was able to rejoin the same job, although per the patient, he was a “changed man”. He was more self-assured, and while he was previously intimidated by his mother-in-law, upon returning, that attitude had changed. He considers himself to have “grown up”. He was more involved in political debates and expressed his opinions fearlessly. He would engage in arguments with his siblings and neighbors from time to time. The patient retired 10-15 years ago, although the specific activities he engaged in post-retirement were not explored in detail. According to collateral information, the patient had become increasingly irritable over the past three years, though his baseline behavior prior to the initial diagnosis in 2014 was not investigated.

Intervention in the unit

A thorough workup was conducted, including a comprehensive metabolic panel, drug abuse screen, heavy metal panel, thyroid function tests, HIV and syphilis screening, and assessment of vitamin levels (Tables 1, 2). All the results were within normal limits, except that arsenic levels were 14 µg/l, which were slightly elevated, and vitamin D 25 hydroxy levels were 30ng/ml, which were slightly lower. The Montreal Cognitive Assessment score was 28/30, with a deficiency in delayed recall (scored 3 out of 5). No other testing was conducted to assess bipolar disorder.

**Table 1:** Drug abuse screen: urine

Amphetamines	Negative
Benzodiazepine	Negative
Cannabinoids	Negative
Cocaine	Negative
Methadone	Negative
Opiates	Negative
Oxycodone	Negative
Fentanyl	Negative

**Table 2:** Heavy metal panel

Arsenic	14 µg/l
Mercury	Not detected
Cadmium	Not detected
Lead	Not detected

On physical and neurological examination, the patient demonstrated good strength and range of motion in all extremities, with no evident tremors or facial drooping. Gait was normal, and no nystagmus was observed. Cardiovascular examination was grossly unremarkable.

Treatment was designed to focus on a supportive and therapeutic environment. He was managed with lamotrigine and olanzapine, targeting bipolar disorder. Lamotrigine was titrated from 100 mg to 150 mg daily, and the olanzapine dose was titrated from 2.5 mg twice daily to 5 mg twice daily during hospitalization. Lamotrigine levels obtained at the time of discharge were 2.3 mcg/ml, which was subtherapeutic. The patient participated daily in various therapeutic activities during hospitalization, including group therapy, dialectical behavior therapy (DBT), recreational therapy, and art therapy. These sessions were conducted on the unit every day, and the patient engaged in them consistently throughout his stay.

Throughout the hospitalization, the patient demonstrated significant improvement in his presenting symptoms. He endorsed improvement in mood and sleep. His mood lability and pressured speech had subsided; he was easily redirectable and not distractible. He engaged in linear, goal-directed conversations without being tangential or circumstantial. He denied any further symptoms, including changes in anhedonia, fatigue, anxiety, active/passive suicidal and/or homicidal ideation, auditory and/or visual hallucinations. A plan to continue treatment with home medications for his chronic medical condition, which included esomeprazole 40 mg before breakfast for GERD, gabapentin 300 mg thrice daily for neuropathic pain, tamsulosin 0.5 mg nightly, verapamil 120 mg nightly, fluticasone nasal spray twice daily, lamotrigine 150 mg daily, and olanzapine 5 mg BID was put in place, and he was encouraged to get psychotherapy and participate in marriage counseling. The patient was discharged on day 9 of his hospitalization.

While we did not follow up with the patient individually after discharge, records indicate that he attended five sessions with his outpatient therapist over a five-week period after discharge. Documentation from these sessions indicated that his mental status examinations (MSE) were within normal limits.

## Discussion

Arnold et al. declared that the elderly represent the fastest-growing group of the population. The age group of >60 years has doubled since 1980, and according to data from developed countries, it is expected to quadruple by 2050, particularly among those >80 years of age. Therefore, it is safe to assume that more patients will be diagnosed with late-onset bipolar disorder while data remains sparse, as large epidemiologic studies primarily focus on adolescents and working-age groups [4].

Therefore, a discussion of cases of late-onset bipolar disorder is crucial. Gathering relevant data and planning for medical care that meets the health needs of this growing population is critical.

This case of late-onset bipolar disorder opens the door to a range of discussions. It highlights the variable presentation in older patients - unexplained by other organic causes and successful management of symptoms - which further solidifies the diagnosis of bipolar disorder, ruling out other possible causes. 

In an older patient, bipolar 1 disorder remains a diagnosis of exclusion following a thorough workup for organic causes, as mentioned in McKenzie et al.: brain tumors, dementia, metabolic disorders, cerebrovascular disease, and medication side effects [5].

Beunders AJM et al. described that depressive and manic symptom severity appears to decrease in old age, which was evident in the case we report. Subtlety of presentation and lack of specific guidelines for late-onset bipolar disorder may lead to misdiagnosis [6]. Further understanding of older age bipolar disorder may lead to more specific recommendations for treatment adjusted to the specific characteristics and needs caused by age-related somatic and cognitive changes [7].

This patient had a history of late-onset bipolar disorder and presented with symptoms of mania on admission. The exact etiology seemed to be unclear, as a very clear timeline couldn't be elicited. From a biological perspective, the patient has the following contributing factors: exposure to Agent Orange, service in the Korean War, a history of head injuries and concussions during his teenage years and prior to his diagnosis of bipolar disorder, and a family history of dementia in his mother.

From a psychological perspective, the patient exhibited immature defense mechanism such as minimization (attempting to reduce the socio-economic impact of his symptoms), acting out (physical and verbal aggression towards family), externalization (blaming spouse and children for his anger and aggression), and rationalization (justifying his lack of need for sleep). This seems to be in line with his poor understanding of his late-onset bipolar disorder and the associated symptoms of impulsivity, flight of ideas, increased energy, rapid speech, and reduced need for sleep.

From a social perspective, the patient’s pattern of relatedness as an adult is characterized by his experiences as a child and in early adulthood. The patient’s formative experiences as defined by the patient were characterized by his deployment to Korea, his experiences with other soldiers, distance from his newly wed wife and child, and stressful experiences while in Korea. This caused strains on his interpersonal relations with his wife and siblings, and later with his children, especially his son.

With this formulation and considering his ongoing treatment, the following management was decided: antipsychotic - olanzapine 5 mg and anticonvulsant - lamotrigine 150 mg BID. This helped target mood reactivity. Group psychotherapy and DBT sessions were encouraged.

Lamotrigine has a good tolerability and safety profile and appears as an “attractive” option for treating bipolar disorder in the elderly [8]. Olanzapine is a highly efficacious second-generation antipsychotic (SGA) utilized for the management of schizophrenia, bipolar disorder, major depressive disorder (MDD) as adjunctive treatment, and agitation associated with schizophrenia or bipolar disorder [9].

According to the literature, a retrospective review that evaluated individuals aged ≥55 years with BP-I who were treated with lamotrigine, lithium, or placebo found that lamotrigine delayed the time to intervention for any mood episode and for a depressive episode when compared to placebo [10]. The use of atypical antipsychotic medications that are approved for the treatment of BD by the FDA in the United States includes aripiprazole, asenapine, olanzapine, quetiapine, quetiapine extended release, risperidone, and ziprasidone [10].

However, although these significantly improved the symptoms of our patient, when further guidelines were searched for, nothing concrete was found.

We found an article published in October 2023, illustrating the literature search conducted using Medline. Literature from 1970 to 2021 was searched using MeSH terms “Bipolar Disorder” x “Aged” or “Geriatric” or “Elderly”, which was complemented by additional literature obtained by observing cross references and by a manual search in textbooks. They referred to old age bipolar disorder as the “orphan” of psychiatric research and further provided a thorough breakdown of the shortcomings related to the available data on bipolar disorder in the elderly [11].

According to the WHO, by 2030, one in 6 people will be above 60 years of age [12]; therefore, gathering relevant data and planning for medical care that meets the health needs of this growing population is imperative. This report aimed to summarize the current state of knowledge about the epidemiology, clinical features, and treatment of bipolar disorder in the elderly. With this case report, we aim to bring to light the need for further research on treatment modalities focusing on pharmacological and psychosocial approaches while not disregarding physical treatments like ECT in elderly patients with BD [11].

## Conclusions

Discussing cases of late-onset bipolar disorder is crucial. As the proportion of older adults in the global population continues to rise, addressing their unique psychiatric needs is critical to future healthcare planning. This demographic shift highlights the necessity for further research on older adults with bipolar disorder (OABD); therefore, gathering relevant data and planning for medical care tailored to meet the health needs of this growing population is imperative. The complexity of the condition in this age group, due to comorbidities, attenuation of symptoms, the anticipated rise in population of the elderly age group (>65) in the coming years, and its public health implications reflect a pressing need for a specialized, standardized, age-sensitive approach to treatment and management.
